# The Impacts of Unfolded Protein Response in the Retinal Cells During Diabetes: Possible Implications on Diabetic Retinopathy Development

**DOI:** 10.3389/fncel.2020.615125

**Published:** 2021-02-03

**Authors:** Kaiser Alam, Yusuf Akhter

**Affiliations:** Department of Biotechnology, School of Life Sciences, Babasaheb Bhimrao Ambedkar University, Lucknow, India

**Keywords:** unfolded protein response, diabetic retinopathy, retinal vascular cell, retinal glial cell, retinal pigment epithelium, retinal neuronal cell

## Abstract

Diabetic retinopathy (DR) is a vision-threatening, chronic, and challenging eye disease in the diabetic population. Despite recent advancements in the clinical management of diabetes, DR remains the major cause of blindness in working-age adults. A better understanding of the molecular and cellular basis of DR development will aid in identifying therapeutic targets. Emerging pieces of evidence from recent research in the field of ER stress have demonstrated a close association between unfolded protein response (UPR)-associated cellular activities and DR development. In this minireview article, we shall provide an emerging understating of how UPR influences DR pathogenesis at the cellular level.

## Introduction

In the worldwide-rising diabetic population, diabetic retinopathy (DR) remains one of the major eye diseases leading to visual impairment and in severe cases even blindness. DR causes noticeable socioeconomic costs for the family of patients and the healthcare system because it significantly affects the working-age population. Epidemiological studies have suggested that about one-third of diabetic patients have some degree of DR and about 10% of them have severe forms of DR, such as diabetic macular edema (DME) and proliferative diabetic retinopathy (PDR). The current DR treatments, such as laser therapy, anti-vascular endothelial growth factor (VEGF) agents, and corticosteroids, usually lead to adverse side effects and are applied only in patients with severe forms of DR (Antonetti et al., 2012; Jenkins et al., 2015).

Besides carrying out many important cellular functions, such as lipid synthesis, calcium ions (Ca^2+^) homeostasis, and storage, the endoplasmic reticulum (ER) is involved especially in protein homeostasis, proteostasis, of membrane and secretory pathway-associated proteins that account for at least one-third of the proteins that are synthesized in the higher organism (Zhang and Kaufman, 2008; Hotamisligil, 2010; Walter and Ron, 2011). To ascertain quality control of protein folding, eukaryotic organisms regulate ER–protein processing activities of misfolded/unfolded proteins at the cellular level known as unfolded protein response (UPR). The activation of UPR results in several outcomes, such as attenuation of global protein translation, enhanced degradation of ER luminal unfolded or misfolded protein, and increased amount of ER-localized chaperones (Rao et al., 2004). Thus, UPR tries to restore ER homeostasis that is essential for cell survival. However, if ER stress is chronic and severe because of unrestorable aggregation of unfolded proteins, UPR induces cell death (Zhang and Kaufman, 2008). Studies in the area of ER stress have implicated dysfunctional UPR on the onset and progression of many diseases including diabetes and its associated complications in various organs, such as the kidney (diabetic nephropathy), neurons (diabetic neuropathy), and eyes (DR; Hotamisligil, 2010).

The retina of the eye is a light-sensitive, greatly complex, and highly organized neural tissue. Recent insight into the physiology of the retina suggests that proper functioning of the retina depends on the close association between neural, glial, and specialized vasculature as a neurovascular unit or neurovascular coupling (Alarcon-Martinez et al., 2020). Communications between different types of the cells in such a neurovascular unit inside the retina are essential for efficient visual function. The retina is considered as an immune-privileged tissue, which is mainly maintained by the blood–retinal barrier (BRB) that safeguards the retinal tissue functions from the systemic immune responses. The BRB prevents easy access of blood constituents to the retina by the complexes of tight junctional (TJ) proteins at two levels, first, between endothelial cells of retinal capillaries that nourish the inner retina (inner BRB) and second, between retinal pigment epithelium (RPE) cells that maintain a barrier for choroidal capillaries nourishing the outer retina (outer BRB; Cunha-Vaz et al., 2011). It has been well documented that prolonged diabetes deteriorates the tiny blood vessels of the eye leading to the breakdown of the BRB. In severe cases, small blood capillaries get occluded to cause ischemia, hypoxia, and finally neovascularization. The advanced and vision-threatening stage of DR is known as PDR, which is characterized by retinal growth of new blood vessels (neovascularization) and epiretinal membrane at the vitreous surface of the retina. These pathological changes can cause vitreous hemorrhage and tractional retinal detachment resulting in the severe loss of vision (Maugh, 1976). Clinical findings and assessment of different stages of DR are based mainly on compromised vasculature in the retina (Antonetti et al., 2012; Jenkins et al., 2015). As DR remains clinically asymptomatic until significantly advanced, new diagnostic tools and therapeutic strategies for the initial stages of its development are urgently needed. In this scenario, the UPR pathways are extremely promising targets to prevent and treat DR at the early stages of its development.

**Figure 1:**
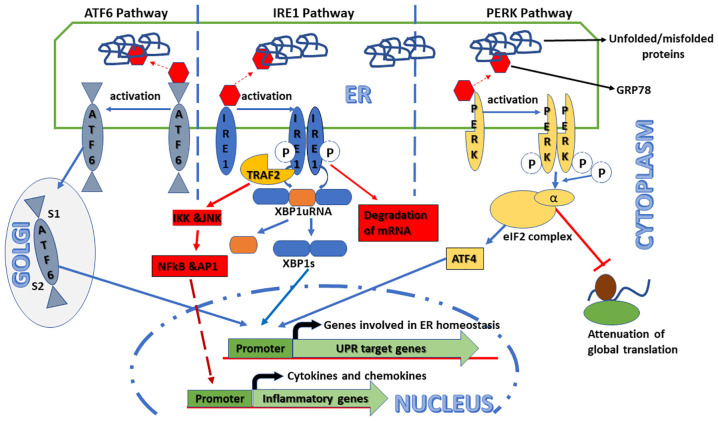
Schematic representation of the signaling pathways activated in the unfolded protein response (UPR). Endoplasmic reticulum (ER) stress can trigger UPR that consists of at least three independent pathways identified by their transmembrane sensor proteins, namely, activating transcription factor 6 (ATF6), inositol requiring enzyme 1 (IRE1), and double-stranded RNA-activated protein kinase (PKR)-like ER kinase (PERK). Under normal conditions, these sensor proteins are inactive because of their association with an ER-resident chaperone protein, 78-kDa glucose-regulated protein (GRP78). However, in ER stress conditions, GRP78 preferentially binds to unfolded/misfolded proteins in the ER lumen leading to the release and activation of the sensor proteins that, in turn, induce their respective downstream signaling pathways. In the ATF6 pathway, GRP78-released ATF6 moves to the Golgi apparatus and is cleaved by both serine proteases and metalloproteases at S1 and S2 sites, respectively. Cleaved ATF6 acts as a transcription factor that binds to the promoters of UPR target genes, such as GRP78 and X-box binding protein 1 (XBP1), and upregulates their expression. In the IRE1 pathway, after its autophosphorylation and oligomerization, GRP78-released IRE1 processes the XBP1 mRNA to generate spliced XBP1 (XBP1s) that, in turn, induces UPR target genes to restore ER homeostasis. Additionally, activated IRE1 also induces the expression and secretion of inflammatory cytokines, such as tumor necrosis factor-α (TNF-α) and interleukin-1β (IL-1β), through the TNF receptor-associated factor 2–IκB kinase–c Jun N-terminal kinase (TRAF2–IKK–JNK) signaling axis. Under some circumstances, it can rapidly degrade some specific subset of mRNAs. In the PERK pathway, after its autophosphorylation and oligomerization, GRP78-released PERK globally attenuates mRNAs to protein translation by phosphorylating the α-subunit of translational initiation factor (eIF2) complex. Additionally, increased eIF2α phosphorylation also modulates the pro- and anti-apoptotic pathways by activating the alternative transcription factor 4-C/EBP homologous protein (ATF4–CHOP) signaling axis.

UPR is known to be a group of phylogenetically conserved intracellular signaling responses and has at least three parallelly or sequentially operated pathways with unique mechanisms of action (Figure 1). Some excellent recent reviews have been published on this subject in the context of various pathophysiological conditions including ocular diseases (Hetz et al., 2013; Zhang et al., 2015; Grootjans et al., 2016; Hetz and Saxena, 2017; Kroeger et al., 2019). Each pathway is named after its ER transmembrane protein, namely, activating transcription factor 6 (ATF6), inositol requiring enzyme 1 (IRE1), and double-stranded RNA-activated protein kinase (PKR)-like ER kinase (PERK). These transmembrane proteins act as sensors of ER luminal unfolded or misfolded proteins. Under normal conditions, 78-kDa glucose-regulated protein (GRP78), a heat shock family-related chaperone protein, binds these sensor proteins in their ER luminal domain, keeping them inactive (Bertolotti et al., 2000). However, during various stress conditions, GRP78 preferably binds to unfolded or misfolded proteins rather than the sensor proteins in the ER lumen, which leads to the activation of the UPR pathways. The downstream signaling of the GRP78-released sensors is well studied and is found to be different within each of the three UPR pathways. In the ATF6 pathways, GRP78-released ATF6 moves to the Golgi apparatus where it is cleaved at different sites by a serine protease and a metalloprotease. Cleaved ATF6 is an active transcription factor that enters the nucleus and activates the promoter of its cognate target genes, such as calreticulin, GRP78, and 94-kDa glucose-regulated protein (GRP94). On the other hand, GRP78-released IRE1, as well as PERK, is activated only after autophosphorylation and oligomerization (Ron and Walter, 2007). In the IRE1 pathway, activated IRE1 acts as a dual activity protein with both kinase and ribonuclease functions. Activated IRE1 removes the internal 28 base pairs segment from X-box binding protein 1 (XBP1) mRNA, generating an alternative splice variant protein XBP1s (Zhang et al., 2015). XBP1s is a transcription factor that either alone or in conjunction with ATF6α upregulates the expression of various ER-localized chaperones and proteins involved in ER-associated protein degradation (ERAD; Lee et al., 2003). It can also rapidly degrade some specific subset of mRNAs depending on their sequence and localization (Hollien and Weissman, 2006). It is pertinent to note that the IRE1 pathway is conserved from lower eukaryotes to higher mammal organisms. Finally, in the PERK pathways, GRP78-released PERK after its autophosphorylation and oligomerization suppresses the activity of translational initiation factor 2 (eIF2) complex by phosphorylating its α subunit. This change in the eIF2 complex not only attenuates the rate of global translation initiation and protein synthesis but also favors the translation of some mRNA, such as activating transcription factor 4 (ATF4; Blais et al., 2004). ATF4 protein is a versatile transcription factor paradoxically involved in both cell survival and apoptosis dependent on the state of ER stress. During acute ER stress, ATF4 induces the genes responsible for adaption; however, under chronic ER stress condition, it promotes apoptosis by upregulating genes, such as CCAAT enhancer-binding protein homologous protein (CHOP; Rozpedek et al., 2016). Additionally, CHOP also contributes to calcium homeostasis in a reactive oxidative stress-dependent manner (Tabas and Ron, 2011). These three pathways either in parallel or in sequence attenuate ER stress.

In recent years, increased mechanistic studies in the field of UPR have suggested that not only different tissues but also different cell types within the organ are represented by different UPR branches (Mori, 2009; Walter and Ron, 2011; Simó and Hernández, 2014; Wang and Kaufman, 2016). However, the activation of the UPR pathways during diabetic conditions in different types of the retinal cells is not properly reviewed, which is the focus of this review.

## Upr Activation and Its Impacts on The Retinal Cells in Dr

In diabetes, chronic hyperglycemia-associated metabolic abnormalities impair the ER functions in many tissues including the retina (Oshitari et al., 2008; Roy et al., 2013). Data from recent research in DR implicate UPR to be activated very early in various retinal cells leading to diabetes-associated cellular dysfunctions and visual impairment.

### Retinal Vascular Cell Dysfunctions

In studies using experimental animal models and patients with diabetes, observed enhanced vascular permeability and neovascularization in DR are closely associated with endothelial dysfunctions and pericyte cell death (Kowluru et al., 2007; Chan et al., 2010; Santos and Kowluru, 2011). The retinal microvascular endothelial cells (RMECs) are one of the principal cells that maintain the inner BRB constituted by TJ complexes composed of transmembrane proteins, such as occludin, claudin, zona occludens, and adhesion molecules (Metea and Newman, 2007). Changes in the expression/activity of these proteins can compromise the integrity of the TJ complex, leading to the breakdown of the BRB, which is a clinically important phenomenon in DR pathogenesis (Klaassen et al., 2009). Several studies have implicated ER stress-mediated retinal inflammation in vascular dysfunctions associated with the development of DR (Adachi et al., 2011). For example, Adachi and colleagues demonstrated that the permeability of externally added fluorescein isothiocyanate (FITC) dextran was significantly increased in the human RMECs treated with ER stress-inducing drugs, such as thapsigargin and tunicamycin. Similarly, these drugs can reduce transendothelial electrical resistance and levels of claudin 5 of TJ complex protein in the RMEC (Adachi et al., 2012).

In DR, enhanced permeability and neovascularization is shown to be critically mediated by VEGF and inflammatory cytokines/chemokines. Several studies have suggested that the activation of UPR regulates VEGF expression and secretion. Li and colleagues reported that in the cultured human RMECs, hypoxia-induced and tumor necrosis factor (TNF)-α expressions were parallelly associated with the activation of UPR with enhanced GRP78, ATF4 levels, and the phosphorylation of IRE1, as well as eIF2α. They also demonstrated that 4-phenylbutyric acid (PBA), a chemical chaperone and attenuator of ER stress, ameliorated these activities (Li et al., 2009). Using rat retinal capillary EC line (TR-iBRB), Chen et al. (2012) showed that high glucose-induced inflammatory cytokines and VEGF expressions were mediated by the activation of the PERK pathway through signal transducer and activator of transcription 3 (STAT3) signaling. Furthermore, Liu et al. (2013) reported that in the human RMECs, diabetic-mimetic conditions increased the expression levels of XBP1/IRE1, ATF6, and a heat shock chaperone α-crystallin B (CRYAB) that consequently upregulate VEGF and knockdown of any of them (IRE1a, ATF6, or CRYAB) by siRNA degrade VEGF (Liu et al., 2013). Recently, Lenin et al. demonstrated that in the human retinal endothelial cell (HREC), hyperglycemia plus TNF treatment decreased transendothelial resistance and the expression of vascular endothelial (VE)-cadherin, which was rescued by ER stress inhibitor drug tauroursodeoxycholic acid (TUDCA; Lenin et al., 2018).

Pericytes are mural cells that partially cover all the blood vessel capillaries. The density of pericytes in retinal capillaries is higher than that in other locations, which suggests their important functional role in the retina. In the adult retina, pericytes are terminally differentiated; therefore, they do not replicate at this site, and their degeneration results in thickening of the basement membrane, increased permeability, and edema (Mandarino, 1992). DR is the only known disease that is strongly correlated to pericyte loss. The pericytes deficiency in the retinal capillaries can lead either to endothelial cell proliferation resulting in microaneurysm (Hammes et al., 2002) or to their degeneration resulting in acellular capillaries, one of the earliest morphological signs of vascular abnormalities in DR. It is worthy to note here that the measurement of acellular capillaries is one of the most reliable markers in experimental DR stages in an animal model.

Several reports implicated UPR activation in retinal pericytes exposed to a fluctuated concentration of glucose. For instance, the UPR pathways were strongly activated not by hyperglycemia but by hypoglycemia or no glucose at all in retinal pericytes leading to their apoptosis (Ikesugi et al., 2006). Similarly, expression levels of transcription factor ATF4 of the PERK pathway of UPR and its target genes CHOP and monocyte chemoattractant protein 1 (MCP-1), an inflammatory molecule, were increased by intermittent high glucose but not by constant high glucose. These glucose fluctuated activities in pericytes were attenuated by a chemical chaperone (Zhong et al., 2012b). In contrary to the above findings, using conditionally immortalized rat retinal pericyte, one report suggests that GRP78 activation and apoptosis were induced by total depletion rather than fluctuations of cellular glucose levels (Makita et al., 2011).

One of the important risk factors for DR onset in type 1 diabetic patients is the increased serum levels of the complexes of oxidatively modified low-density lipoprotein (oxLDL) and its auto-antibodies, oxLDL immune complexes (Ox-LDL-IC; Lopes-Virella et al., 2012). Fu et al. (2014) demonstrated that Ox-LDL-IC was cytotoxic to retinal pericytes. Ox-LDL-IC-induced apoptosis of pericytes was mediated also by ER stress, which was triggered by scavenger and IgG receptors. Recently, it was reported that advanced glycation end product (AGE) or modified low-density lipoprotein induced the PERK pathway of UPR in human retinal pericytes, which was ameliorated by a chemical chaperone, ursodeoxycholic acid (UDCA; Chung et al., 2017).

### Retinal Glial Cell Dysfunctions

The retinal glial cells, also considered as innate immune cells of the retina, are composed of three types, namely, astrocytes, microglia, and Müller cells (MCs). They are the main producer of proinflammatory cytokines, such as TNF-α, interleukin (IL)-1β, and IL-6 (Yang et al., 2008; Kochan et al., 2012). The predominant glial cell types in the retina are MCs that span all across the retina from the outer limiting membrane to the inner limiting membrane (Newman and Reichenbach, 1996; Ahmad et al., 2011). MCs are involved in many critical retinal functions, such as ion homeostasis regulation, structural stabilization, neuronal survival, and neurotransmitter recycling (Reichenbach et al., 1993; Chan-Ling, 1994; Kim et al., 2006). Furthermore, they are involved in providing trophic and anti-oxidative support to neurons and maintaining the BRB. MCs function as a soft protector for neurons, keeping them safe from external injuries, such as mechanical trauma. They are proposed to be living optical fibers that transduce light through the internal neuron, preventing the scattering of light thereby enhancing signal/noise ratio (Reichenbach and Bringmann, 2013). During stress conditions, MCs absorb glucose and nutrients from the blood and store them as glycogen that can be utilized as an energy source. They also uptake and detoxify glutamate and provide glutamine and lactate as metabolites to neurons (Zahs et al., 2003; Zahs and Ceelen, 2006). Furthermore, MCs remove retinal fluid to prevent retinal edema, which is an extremely important function because the retina does not have a lymphatic drainage system (Zhao et al., 2010, 2011). However, when retinal homeostasis is disturbed due to various pathophysiological conditions, such as diabetes, MCs get activated and undergo reactive gliosis (Bringmann et al., 2009).

The activation of MCs was found to be one of the main factors for DR onset and progression (Feit-Leichman et al., 2005; Wong et al., 2011), which is crucially dependent on the activation of the UPR pathways and the expression/secretion of VEGF and inflammatory cytokines. For example, Zhong et al. (2012a) reported that high glucose exposed MCs upregulated the expression of VEGF and the activation of ATF4, a PERK pathway transcription factor. Using rat MC line, Wu et al. (2019) recently reported that both hyperglycemia and its osmotic control mannitol activated the UPR pathways to upregulate the expression of VEGF. They demonstrated that all three pathways of UPR were interdependent on one another for their VEGF upregulation function. Recently, Yang and colleagues have demonstrated that primary MCs derived from genetically Xbp1 knockout (Xbp1 Müller−/−) mice expressed higher levels of VEGF, TNF-α, phosphorylated c-Jun N-terminal kinase (p-JNK), and ER stress markers (except GRP78) and intensified inflammation and ER stress when exposed to high glucose or hypoxia than MCs derived from control mice. They further demonstrated that the chemical chaperones both PBA and trimethylamine N-oxide (TMAO) suppressed glucose or hypoxia-induced VEGF and TNF-α from Xbp1 knockout MCs (Yang et al., 2019).

### RPE Cell Dysfunctions

The RPE cells are pigmented in nature and constitute the outer BRB of the retina. Its long apical microvilli enclose a light-sensitive neural segment of the retina. The basolateral part of the RPE cells touches the Bruch’s membrane, which separates choriocapillaris fenestrated endothelium (Strauss, 2005). The RPE cells are involved in many activities important for proper visual functions, such as the transformation of all-trans-retinal into 11-cis-retinal, necessary for visual cycles, shed photoreceptor membrane phagocytosis, extra-light absorption, and protection from photooxidation (Simó et al., 2010). Although relatively fewer studies on DR focus on the RPE cells than on the RMECs for DR-associated dysfunctions, such as vascular leakage and changes on TJ proteins, they indeed suggested that diabetes damages the RPE cells of the retina (Kirber et al., 1980; Vinores et al., 1989; Xu and Le, 2011).

Several studies reported that the expression and secretion of VEGF in diabetic retinal RPE cells were mediated by the activation of the UPR pathways (Zhang et al., 2015; Salminen et al., 2010). One group reported that homocysteine-induced VEGF in the RPE cells was mediated by the ATF4 transcription factor of the PERK pathway (Roybal et al., 2004). Similarly, the phosphorylation of eIF2α indicating the activation of the PERK pathway is reported in the RPE cells of patients with the non-proliferative stage of DR (Miranda et al., 2012). Du et al. (2013) demonstrated that the RPE cells of diabetic patients with retinopathy had more levels of GRP78 chaperone than those of diabetic patients without retinopathy, suggesting the involvement of UPR of the RPE cells in DR development.

Additionally, several traditional herbal drugs were shown to ameliorate diabetic-mimetic condition-induced UPR activation in the RPE cells. It was reported that beta-glucogallin, an aldose reductase inhibitor isolated from Indian gooseberry fruit, was shown to ameliorate GRP78 induced by hyperglycemia in the RPE cells (Chang et al., 2015). Furthermore, in the ARPE-19 cell line, grape polyphenol was shown to attenuate the thapsigargin-induced activation of the UPR pathways, VEGF expression, and apoptosis (Ha et al., 2014). Kang et al. (2018) demonstrated that in the RPE cells, chrysin, a flavone-type flavonoid, attenuated diabetic-mimetic conditions, or ER stress inducer drugs mediated the induction of ATF6, IRE1α (Kang et al., 2018). Recently, Peng et al. (2020) demonstrated that in the RPE cells, astragalus polysaccharide, a traditional Chinese medicine, ameliorates UPR activation, as well as apoptosis, *via* the regulation of the miR-204/SIRT1 signaling axis in an *in vitro* metabolic memory model (Peng et al., 2020).

### Retinal Neuronal Cell Dysfunctions

The neural retina is composed of many cell types, such as rods, cones, and horizontal, bipolar, amacrine, and ganglion cells. Although the first clinical signs of DR are vascular changes, diabetes can damage the neuronal retina resulting in alterations in retinal functions (Shirao and Kawasaki, 1998; Li et al., 2002; Phipps et al., 2006). Among the retinal neuron, the retinal ganglion cells (RGCs) are widely investigated concerning the effect of diabetes (Kern and Barber, 2008). Several reports have demonstrated RGCs loss in the retina of diabetic patients or experimental animal models (Barber et al., 1998; Martin et al., 2004; Oshitari et al., 2008).

Cell death of the ganglion cells was proposed to be a relatively early phenomenon in DR, which is dependent on the activation of the UPR pathways. Oshitari et al. (2011) reported that apoptosis of RGCs from the diabetic retina or non-diabetic retina *in vitro* exposed to high glucose correlated the activation of the PERK pathway with increased levels of CHOP. Using an STZ-induced diabetic animal model, Yang and colleagues reported that although CHOP of the PERK pathways was significantly increased all along with the nerve fiber and ganglion cell of the retina, JNK and caspase 12 protein, molecules critically involved in apoptosis, were found to be increased and restricted in the ganglion cell layer. They also reported that the intraperitoneal injection of the drug TUDCA ameliorated levels of all these molecules, as well as DR-associated dysfunctions (Yang et al., 2013). Using the transformed mouse RGC cell line (mRGC), Zhang et al. (2018) demonstrated that ganglion cell apoptosis in the context of DR was mediated by PERK pathways activation through ATF4 and CHOP, which is ameliorated by a Chinese herbal medicine He-Ying-Qing-Re Herbal Formula (HF).

## Summary and Conclusion

Although the pathogenesis of DR is extremely involved, it is becoming clearer that the UPR can either protect the diabetic retina from retinopathy or promote it to the initiation, progression, and aggravation of retinopathy. Early events of DR pathogenesis include gliosis of MCs, apoptosis/degeneration of vascular (pericytes), and neuronal (ganglion) cells, precipitating vascular abnormalities. Research in the field of DR has provided a body of evidence indicating the activation of the UPR pathways in most of the retinal cells. In the RMECs and RPE cells, DR condition-exposed increased VEGF and cytokine expressions are reported to be mediated by the PERK and IRE1 pathways of UPR. Moreover, diabetic-mimetic conditions mediated RGC and pericytes cell death are demonstrated to be medicated by the PERK pathways through CHOP and ATF4. Furthermore, all three pathways of UPR are reported to be necessary for VEGF and cytokines mediated MCs activation. Additionally, several chemical and herbal drugs were reported to ameliorate diabetic-mimetic condition-induced UPR activation and DR development.

**Figure 2:**
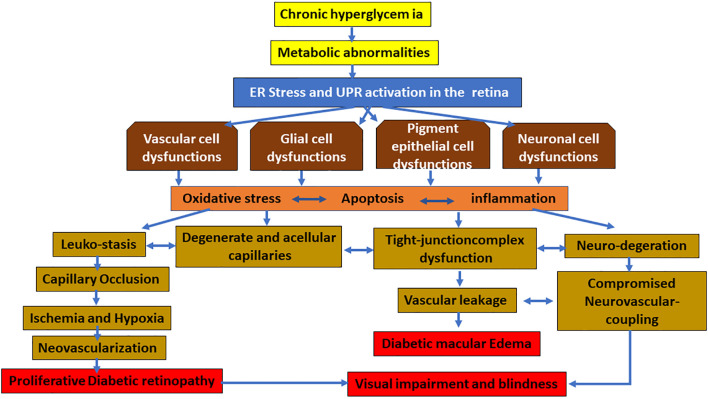
Hypothesized schematic representation of the UPR pathways causing damage to the vascular, glial, pigment epithelial, and neuronal cells of the retina in diabetic retinopathy (DR). In diabetes, chronic hyperglycemia and its associated metabolic abnormalities lead to ER stress in the retinal cells. While temporary and mild ER stress can be overcome by the adaptive UPR, persistent ER stress and the consequent UPR activation can follow different pathways to activate the proinflammatory and proapoptotic signaling pathways. This results in damage to various cells of the retina. The characteristic changes of early diabetic retinas may be glial cell activation, which, in turn, leads to vascular changes leading to apoptosis and dropout of pericyte, as well as dysfunctional tight junctional complex proteins of endothelial and pigment epithelial cells resulting in the inner and outer blood–retinal barrier (BRB) breakdown, respectively. Furthermore, neuronal abnormalities lead to compromised neurovascular coupling and finally neovascularization and hemorrhage, resulting in vision-threatening DR such as macular edema and proliferative diabetic retinopathy.

However, it is not clear how different pathways of UPR in different types of the cells are temporally and spatially interrelated and altered in the neurovascular unit of the retina during diabetes. Published studies suggest that the early activation of UPR in the glial cells, such as MCs, triggers the proinflammatory and/or proangiogenic pathways in an oxidative stress-dependent manner resulting in the expression and secretion of various neurodegenerative, proinflammatory, and proangiogenic growth factors, cytokines, and chemokines, which, in turn, lead to vascular abnormalities observed in PDR (Figure 2). Additional studies are needed for deeper understanding and determining the best preventive and therapeutic target of DR in the UPR pathways.

## Author Contributions

KA conceived and designed the work, analyzed the published data, wrote, critically reviewed and revised the manuscript. YA analyzed the published data, critically reviewed and revised the manuscript. Both authors contributed to the article and approved the submitted version.

## Conflict of Interest

The authors declare that the research was conducted in the absence of any commercial or financial relationships that could be construed as a potential conflict of interest.
